# Cholinergic muscarinic receptor activation augments murine intestinal epithelial cell proliferation and tumorigenesis

**DOI:** 10.1186/1471-2407-13-204

**Published:** 2013-04-24

**Authors:** Zhongsheng Peng, Jonathon Heath, Cinthia Drachenberg, Jean-Pierre Raufman, Guofeng Xie

**Affiliations:** 1 Division of Gastroenterology and Hepatology, Veterans Affairs Maryland Health Care System, University of Maryland School of Medicine, Baltimore, MD, USA; 2 Department of Pathology, University of Maryland School of Medicine, Baltimore, MD, USA

**Keywords:** Bethanechol, Cholinergic agonist, Cell proliferation, Colon cancer, Matrix metalloproteinase

## Abstract

**Background:**

Previously, we showed that M3 muscarinic receptor (M3R; gene name *Chrm3*) deficiency attenuates murine intestinal neoplasia, supporting the hypothesis that muscarinic receptors play an important role in intestinal tumorigenesis.

**Methods:**

To test this hypothesis, in the present study we treated mice with bethanechol, a non-selective muscarinic receptor agonist without nicotinic receptor activity, and examined its effects on azoxymethane (AOM)-induced colon neoplasia. Mice were provided with drinking water containing 400 μg/mL bethanechol chloride or water without additions (control) for a total of 20 weeks, a period that included the initial 6 weeks when mice received intraperitoneal injections of AOM.

**Results:**

When euthanized at week 20, control mice had 8.0 ± 1.3 tumors per animal, whereas bethanechol-treated mice had 10.4 ± 1.5 tumors per mouse (mean ± SE; P = 0.023), a 30% increase. Strikingly, tumor volume per animal was increased 52% in bethanechol-treated compared with control mice (179.7 ± 21.0 vs. 111. 8 ± 22.4 mm^3^; P = 0.047). On histological examination, bethenechol-treated mice also had more adenocarcinomas per animal (8.0 ± 1.0 vs. 4.1 ± 0.6 for control mice, P = 0.0042). Cell proliferation in both normal mucosa and adenocarcinomas was increased in bethanechol-treated compared to control mice. Also, in tumors, bethanechol treatment increased expression of *Chrm3*, *Egfr* and post-*Egfr* signaling molecules *Myc* and cyclin D1. Bethanechol treatment increased the thickness of normal colonic mucosa and the expression of selected matrix metalloproteinase (*Mmp*) genes, including *Mmp7*, *Mmp10* and *Mmp13*.

**Conclusions:**

These findings support a prominent role for muscarinic receptors in colon neoplasia, and identify post-receptor signaling molecules as potential therapeutic targets.

## Background

The muscarinic cholinergic family of G protein-coupled receptors (GPCRs) consists of five subtypes designated M1R-M5R. Emerging evidence indicates that muscarinic receptors play an important role in colon cancer cell proliferation and tumorigenesis. M1R, M3R and M5R, which are coupled to G_q11_ and activate phospholipase C, are conditional oncogenes when expressed in cells capable of proliferation [1]. In particular, M3R are expressed widely in intestinal epithelium and over-expressed in the majority of human colon cancer cell lines and tumors, up to 8-fold compared to adjacent normal tissue [2,3]. Our previous work showing that reducing M3R expression and activation attenuates intestinal epithelial cell proliferation and neoplasia in both *Apc*^*min/+*^[4] and azoxymethane (AOM) [5] murine models supports a functional role for M3R expression and activation in colon cancer.

Additional biological plausibility for the role of M3R was provided by work from our lab elucidating the molecular mechanisms underlying cholinergic agonist-induced colon neoplasia. Using human colon cancer cells that express high levels of M3R, we showed that muscarinic agonist-induced cell proliferation is mediated by cross-talk between M3R and epidermal growth factor receptors (EGFRs) and activation of post-EGFR signaling mediated by p44/42 mitogen-activated protein kinase (ERK) [6,7]. Also, we showed that M3R activation stimulates robust expression of matrix metalloproteinase (MMP) genes, including *MMP1*, *MMP7* and *MMP10*, which play key roles in mediating cell proliferation, cell migration and invasion *in vitro*[8-10].

Based on these observations, we hypothesized that activation of M3R *in vivo* using a muscarinic receptors agonist would up-regulate MMP gene expression, and enhance cell proliferation and neoplasia. To test this hypothesis, we treated mice with bethanechol (carbamyl-β-methylcholine chloride), a non-selective muscarinic receptor agonist without nicotinic receptor activity, and examined its effects on tumor formation, cell proliferation and *Mmp* gene expression in the AOM mouse model of colon neoplasia. Results from this *in vivo* work reveal the importance of muscarinic signaling for promoting intestinal epithelial cell proliferation and colon neoplasia, and identify a role for selected MMP genes in intestinal tumorigenesis.

## Methods

### Materials

Carbamyl-β-methylcholine chloride (bethanechol) was purchased from Sigma; azoxymethane was purchased from the Midwest Research Institute. All other chemicals were obtained from Sigma-Aldrich or Fisher Scientific.

### Animals

WT mice (129S6/SvEv X CF1) were purchased from Taconic Farms. For all experiments, six-week-old male mice were used. Mice were housed under identical conditions in a pathogen-free room, had free access to commercial rodent chow and water, and were allowed to acclimatize in the vivarium for 2 weeks prior to treatments. Mice were weighed weekly. This study was approved by the Office of Animal Welfare Assurance from the University of Maryland School of Medicine and the Research Development Committee at the VA Maryland Health Care System.

### Bethanechol and azoxymethane (AOM) treatment

Six-week-old WT male mice were randomly allocated to two groups; treatment with AOM was started concurrently with provision of drinking water with or without (control) addition of 400 μg/mL bethanechol. For the initial 6 weeks, animals received weekly intraperitoneal injection of AOM (10 mg/kg body weight). Mice were freely allowed to drink water with or without bethanechol for an additional 14 weeks (total of 20 weeks) and did not exhibit any notable side-effects. Volumes of water consumed by each group of mice were measured weekly and were not significantly different between the two groups throughout the study period. Mice were observed for evidence of tumor formation (e.g., anal bleeding) and euthanized at week 20.

### Tumor measurement and histologic analysis

After euthanasia, colon segments were opened longitudinally and placed flat on microscope slides. Tumors were identified by visual inspection and photographed using a dissecting microscope (Nikon SMZ1500). Tumor diameter was measured using Nikon Image-Pro (Image Pro International, Miami, FL) and tumor volume calculated: volume = 1/2(length × width^2^) [5]. The distal half of colons that contained tumors were fixed in 4% paraformaldehyde and paraffin embedded. Serial five-micron sections were stained with H&E and examined by experienced pathologists masked to treatment. Tumor counts and classification of tumor types were adjudicated by two expert pathologists (JH and CD) who were masked to treatment. Adenomas and adenocarcinomas were defined according to consensus recommendations from the Mouse Models of Human Cancers Consortium [11].

Interstitial inflammation in normal colonic mucosa and tumors was scored by an expert pathologist (CD) using the following grading scale, modified from similar scoring systems for human tissue: 0, Rare mononuclear interstitial inflammation, with no formation of mononuclear cell clusters; 1+, Mild interstitial inflammation with formation of small cell clusters but with no separation of the glands; 2+, Moderate interstitial inflammation with formation of cell clusters that separated the glands, with cell clusters not larger than average normal crypts; 3+, Marked interstitial inflammation with formation of cell clusters that separated the glands, with cell clusters larger than average normal crypts.

### Immunohistologic analysis and quantification

Paraffin-embedded sections were stained with antibodies against mouse Ki67 and cleaved (activated) caspase-3 according to manufacturer’s instructions. Quantification of Ki67 and cleaved caspase-3 staining was performed as described [12]. Briefly, slides were scanned using the Aperio ScanScope Cs (Aperio Technologies, Vista, CA) with a 20× objective. Regions of interests were selected by an experienced pathologist (JH) and quantification of staining was performed using ImageScope software.

### Quantitative real-time PCR (QPCR)

QPCR and quantification of mRNA levels was performed as described previously [10]. Primer sequences for mouse genes used in this study are shown in Additional file 1: Table S1. Specificity of amplifications was confirmed by melting-curve analysis. Relative levels of mRNA were calculated according to the standard ΔΔCt method. Individual expression values were normalized by comparison with glyceraldehyde-3-phosphate dehydrogenase (*Gapdh*).

### Statistical analysis

All data are expressed as mean ± SE of at least three independent experiments. Statistical calculations were performed using Student’s un-paired t-test (SigmaPlot, Systat Software, Inc., San Jose, CA). P < 0.05 was considered significant.

## Results

### Bethanechol promotes colon tumor formation

Previously, we showed that M3 muscarinic receptors (M3R) promote intestinal tumorigenesis in murine models of colon cancer [4,5]. Based on our observations that M3R deficiency and treatment with a muscarinic receptor antagonist, scopolamine butylbromide, reduces intestinal tumor formation [4], we hypothesized that treatment with a muscarinic agonist would promote neoplasia *in vivo*. To test our hypothesis, we examined the effect of bethanechol on tumor formation and growth in the AOM mouse model of colon cancer. Compared to the other commonly-used murine intestinal neoplasia model, *Apc*^*min/+*^ mice which develop primarily small intestinal adenomas, AOM-treated mice develop only colon tumors, including both adenomas and adenocarcinomas, which more closely mimic human colon neoplasia.

For these studies, we chose bethanechol, a non-selective muscarinic agonist because it lacks nicotinic receptor activity, is commonly used in murine studies of salivation and neurological function [13-15], does not cross the blood–brain barrier, has excellent short- and long-term safety profiles in mice [16,17], and is FDA-approved (trade name Duvoid) for treatment of urinary retention and atony of the urinary bladder. As anticipated from our previous work, bethanechol increased cell proliferation in H508 human colon cancer cells that express primarily M3R (data not shown).

As described in Materials and Methods, we fed mice with either drinking water containing no additions (control) or 400 μg/mL bethanechol chloride for a total of 20 weeks, a duration chosen based on our previous experience with AOM as an inducer of colon tumors [5]. For the initial 6 weeks, mice also received weekly intraperitoneal AOM injections. Based on water consumption records, on average, mice in each group drank similar amounts of water (30 mL/wk at week 1 to 40 mL/wk at week 20); these volumes were consistent with consumption of 12 to 16 mg bethanechol per week in the treatment group. Bethanechol treatment did not result in any noticeable side effects.

**Figure 1:**
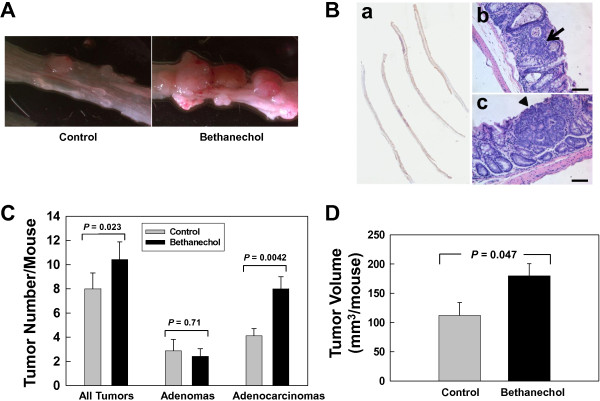
**Treatment with bethanechol increases AOM-induced murine colon tumor number and volume. A**. Representative dissecting microscopic images of AOM-induced colon tumors from mice that were fed water (control; *N* = 8) or water containing bethanechol (*N* = 7). **B**. Representative H&E-stained sections (magnification, 200×). Size bars represent 100 microns. a. Representative scanned image of entire distal half of colon that contains tumors. b. Arrow indicates an adenoma. c. Arrowhead indicates an adenocarcinoma. **C**. Effect of bethanechol treatment on tumor number. **D**. Effect of bethanechol treatment on tumor volume. Bars in **C** and **D** represents mean ± SE.

At week 20, mice in each group gained a similar percentage of initial body weight (control vs. bethanechol; 31% vs. 32%; Additional file 2: Figure S1, panel A). In addition, at week 20, colon length in animals in each group was not significantly different (13.5 cm ± 1.0 cm vs. 13.4 cm ± 0.9 cm, control compared to bethanechol; P = 0.79; Additional file 2: Figure S1, panel B). As shown in Figure 1, compared to control, 20 weeks of bethanechol treatment significantly increased tumor numbers by 30% (10.4 ± 1.5 vs. 8.0 ± 1.3; P = 0.023) and tumor volume by 52% (179.7 ± 21.0 vs. 111.8 ± 22.4 mm^3^; P = 0.047). The increase in tumor number was primarily a consequence of increases in numbers of adenocarcinomas (8.0 ± 1.0 vs. 4.1 ± 0.6; P = 0.0042). Collectively, these findings indicate that in this murine model of colon cancer, bethanechol treatment increases the size and number of tumors, more specifically the number of adenocarcinomas.

### Bethanechol stimulates cell proliferation and increases mucosal thickness

**Figure 2:**
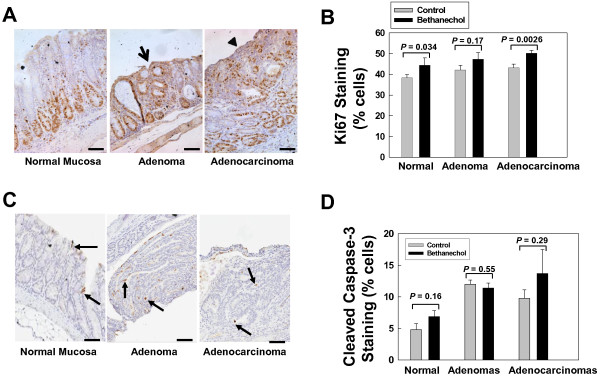
**Markers of cell proliferation and apoptosis in normal colonic mucosa and tumors from each treatment group. A**. Representative Ki67-stained sections (magnification, 200×). Arrow indicates an adenoma. Arrowhead indicates an adenocarcinoma. Size bars represent 100 microns. **B**. Percentage of Ki67-positive cells in normal colon mucosa, adenomas and adenocarcinomas. **C**. Representative cleaved caspase 3-stained sections. Arrows indicate cleaved caspase 3-positive cells. **D**. Percentage of cleaved caspase-3-positive cells in normal colon mucosa, adenomas and adenocarcinomas. Bars in **B** and **D** represents mean ± SE. *N* = 8 and 7 for control and bethanechol-treated groups, respectively.

**Figure 3:**
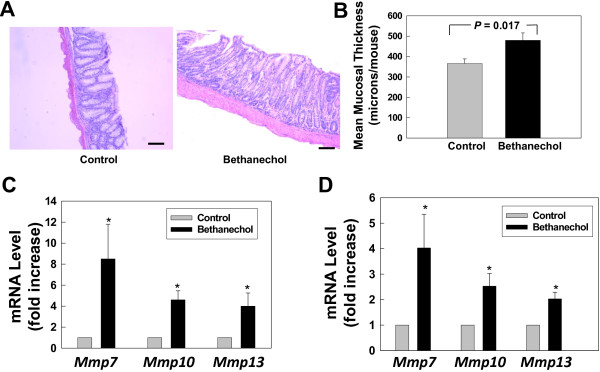
**Effect of bethanechol treatment on colon mucosal thickness and expression of matrix metalloproteinase genes. A**. Representative H&E-stained normal colon mucosa from control and bethanechol-treated mice. Size bars represent 100 microns. **B**. Intestinal mucosal thickness in tissue from control and bethanechol-treated mice. **C**. Expression of *Mmp* mRNAs in normal proximal colon mucosa from each treatment group. **D**. Expression of *Mmp* mRNAs in tumors from each treatment group. QPCR was performed as described in Materials and Methods. Results are expressed as mean ± SE from 5 samples from each group. *P < 0.05, Student’s un-paired *t*-test.

To determine whether bethanechol-induced increased tumor formation results from increased cell proliferation, reduced apoptosis or a combination of both, we examined tumors for changes in markers of cell proliferation and apoptosis, using Ki67 and cleaved caspase-3 staining, respectively. As shown in Figure 2, bethanechol treatment increased cell proliferation in both normal epithelium (44.4% ± 3.5% vs. 38.3% ± 1.6%; P = 0.034) and adenocarcinomas (50.2% ± 1.4% vs. 43.2% ± 1.8%; P = 0.0026), whereas apoptosis was not significantly altered (although there was trend towards increased cleaved caspase-3 staining in adenocarcinomas from bethanechol-treated mice; Figure 2D). Also, as shown in Figure 3A, B, bethanechol treatment increased colon mucosal thickness (479.9 ± 36.3 microns vs. 365.9 ± 22.8 microns; P = 0.017). These results suggest that bethanechol promotes tumor formation primarily by stimulating cell proliferation, a finding consistent with the striking increase in tumor size (Figure 1D).

### Bethanechol up-regulates expression of matrix metalloproteinase (Mmp) genes

Previously, in human colon cancer cell lines, we showed that of the ~25 known MMP genes, acetylcholine treatment selectively induced robust transcription of *MMP1*, *MMP7* and *MMP10*[10]. We also showed that ACh-induced *MMP1* and *MMP7* expression promotes cell proliferation, cell migration and invasion *in vitro*[8-10]. Based on these findings, we hypothesized that bethanechol treatment in mice would induce expression of specific *Mmp*s, mimicking the *in vitro* data in human colon cancer cells. To test this hypothesis, in AOM-induced colon tumors from mice treated with or without bethanechol, we performed QPCR to examine the expression of murine *Mmp1a*, *Mmp7*, *Mmp10* and other *Mmps* that are also implicated in colon carcinogenesis [18-20], including *Mmp2*, *3*, *9* and *13*. Murine *Mmp1a* is the homologue of human *MMP1*[21].

As shown in Figure 3C, D, in both normal colon mucosa and AOM-induced tumors, bethanechol treatment robustly increased *Mmp7* and *Mmp10* mRNA levels. Although *Mmp1a* was not detected in normal mucosa and tumors (data not shown), bethanechol treatment increased mRNA level of *Mmp13*, which encodes the major murine interstitial collagenase and is therefore a functional homolog of human MMP1. Not surprisingly, the mRNA levels of *Mmp7*, *10* and *13* were much higher in tumors than normal mucosa (data not shown). In contrast, expression of *Mmp2*, *Mmp3* and *Mmp9* was not altered (data not shown).

### Bethanechol stimulates expression of muscarinic receptors

**Figure 4:**
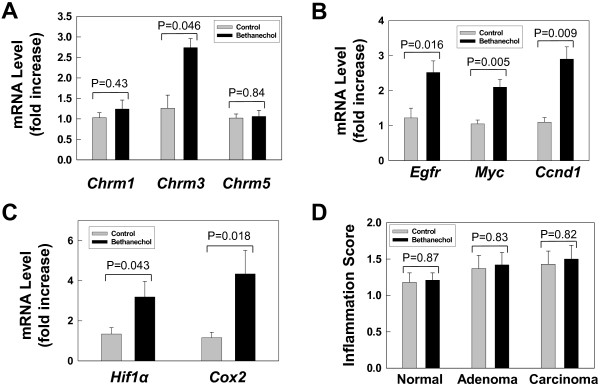
**Effect of bethanechol treatment on tumor gene expression and interstitial inflammation. A**. Expression of muscarinic receptor mRNAs (*Chrm1*, *3* and *5*) in tumors from control and bethanechol-treated mice. **B**. Expression of *Egfr*, *Myc* and *Ccnd1* in tumors from each treatment group. **C**. Expression of *Hif1α* and *Cox2* in tumors from each treatment group. QPCR was performed as described in Materials and Methods. Results are expressed as mean ± SE from 5 samples from each group. **D**. Interstitial inflammation scores of control and bethanechol-treated normal mucosa and tumors (see Materials and Methods). Results are expressed as mean ± SE.

Previously, Song et al. used nicotine-stimulated expression of nicotinic acetylcholine receptors (nAChR) as a measure of activated nAChR signaling [22]. Likewise, we used QPCR to determine whether bethanechol treatment altered expression of muscarinic receptors. As shown in Figure 4A, compared to tumors from control mice, *Chrm3* was over-expressed in tumors from bethanechol-treated mice (2.7-fold; P = 0.046), whereas *Chrm1* and *Chrm5* expression was not affected. *Chrm2* and *Chrm4* were not expressed in either normal colon mucosa or tumors.

### Bethanechol treatment increases expression of Egfr and post-Egfr signaling molecules

Previously, in human colon cancer cells, we showed that cholinergic agonists stimulate cell proliferation by trans-activating EGFR and stimulate post-EGFR signaling. We performed QPCR to determine whether bethanechol treatment caused similar effects in tumors. As shown in Figure 4B, bethanechol treatment stimulated greater than 2-fold increases in expression of *Egfr, Myc and cyclin D1 (Ccnd1)*.

### Bethanechol treatment does not alter interstitial inflammation

Mariani et al. showed that, in human colon adenomas and carcinomas, increased expression of two pro-inflammatory genes, *COX2* and *HIF1α*, strongly correlated with the degree of dysplasia [23]. We used QPCR to determine whether bethanechol treatment altered *Cox2* and *Hif1α* expression. As shown in Figure 4C, in tumors from bethanechol-treated mice, we detected increased mRNA levels for both *Cox2* and *Hif1α*. However, despite these modest increases in *Cox2* and *Hif1α* expression, interstitial inflammation was not different when we compared control and bethanechol-treated normal colon mucosa and tumors (Figure 4D). In addition, although there were trends towards increased inflammation in tumors, these were not statistically significant [i.e., bethanechol-treated adenocarcinomas vs. untreated normal mucosa (P = 0.19)].

## Discussion

Growth factors and their receptors play important roles in promoting cancer cell proliferation and tumor growth. Previously, we showed that M3 muscarinic receptors promote intestinal epithelial cell proliferation and tumorigenesis both *in vitro* in human colon cancer cell lines and *in vivo* in animal models. In the present study, we asked and answered a key question regarding the role of muscarinic agonists as growth factors for colon neoplasia – Do muscarinic receptor agonists promote intestinal neoplasia *in vivo*?

Using the AOM mouse model of colon cancer, we showed that a muscarinic receptor agonist bethanechol promotes intestinal epithelial cell proliferation, increases colonic mucosal thickness and promotes tumor formation. To our knowledge, this is the first report showing that a cholinergic muscarinic agonist stimulates intestinal epithelial cell proliferation and tumorigenesis *in vivo*. This observation further affirms a key role for muscarinic receptors and ligands in colon neoplasia.

As shown in Figure 1C, D, bethanechol treatment not only increased the size but also the number of tumors, primarily by increasing the number of more advanced tumors, i.e., adenocarcinomas. These findings suggest that cholinergic agonist-muscarinic receptor interaction may play a role in both tumor initiation and promotion. This is consistent with our previous observation that in azoxymethane-treated mice, genetic ablation of *Chrm3* attenuates both tumor size and tumor number, including the number of adenocarcinomas [5]. To confirm the role of cholinergic agonists and muscarinic receptors in tumor initiation, future studies will examine the effects of bethanechol treatment on aberrant crypt foci and mucin-depleted foci; both are early markers of colon carcinogenesis [11].

As shown in Figure 4A, bethanechol treatment stimulated expression of *Chrm3* in tumors. This finding suggests that bethanechol directly acts on muscarinic receptors in colonic epithelial cells to promote cell proliferation and tumor growth. Song and colleagues reported similar findings in squamous cell lung carcinomas wherein increased expression of cholinergic receptors and ligands were a result of direct ligand-receptor interaction [22].

As shown in Figure 4B, bethanechol treatment also stimulated expression of *Egfr* and post-*Egfr* signaling molecules *Myc* and *Ccnd1*. Previously, in human colon cancer cells we showed that cholinergic agonist-MR interaction cross-talks with EGFR and activates post-EGFR signaling to promote cell proliferation and tumor invasion [6]. These findings reaffirm an important role for EGFR and post-EGFR signaling in cholinergic agonist-stimulated tumorigenesis *in vivo*.

Chronic mucosal inflammation plays an important role in intestinal carcinogenesis [24]. It has been reported previously that expression of both *COX2* and *HIF1α* were up-regulated in the early steps leading to colorectal carcinogenesis and correlate with the degree of dysplasia [23]. As shown in Figure 4C, D, although bethanechol treatment modestly stimulated expression of two pro-inflammatory genes *Cox2* and *Hif1α*, it did not result in significant differences in interstitial inflammation, suggesting that inflammation does not play a significant role in bethanechol-stimulated tumorigenesis in this AOM animal model of colon carcinogenesis. Nonetheless, there is inherent subjectivity to the method we used to score inflammation in murine colorectal lesions (see Materials and Methods). Hence, we acknowledge that differences between study groups may have been underestimated.

Another thought-provoking observation was that bethanechol treatment increased the thickness of normal colonic mucosa by promoting epithelial cell proliferation. This pro-proliferative action of bethanechol on non-cancerous epithelial cells suggests a possible role in development and may be useful in promoting mucosal healing in intestinal injury and inflammatory bowel diseases.

The extracellular matrix (ECM) plays an important role in normal physiological processes and in carcinogenesis, including cell proliferation, tumor growth, invasion and dissemination [18-20]. In this study we showed that bethanechol stimulated robust expression of specific *Mmp* genes, i.e., *Mmp7*, *Mmp10* and *Mmp13* (Figure 3C, D). Up-regulation of specific *Mmp* genes likely contributed to increased numbers of adenocarcinomas in the bethanechol treatment group. In a systematic analysis of *MMP* gene transcription, we identified the same three *MMP* genes [*MMP1* (functional human homologue of mouse MMP13), *MMP7* and *MMP10*] as target genes for acetylcholine-M3R activation in human colon cancer cell lines [10]. We showed that these three *MMP* genes, which have non-overlapping functions, act together to maximally increase tumor growth and malignant properties of neoplastic cells, including cell migration and invasion. *MMP1* and *Mmp13* encode, respectively, the major collagenase in human and mouse that degrade type I-III interstitial collagens. MMP7 degrades several ECM proteins including elastin and casein, whereas MMP10 primarily degrades proteoglycans and fibronectin. We showed previously that MMP7 also acts as a growth factor/signaling molecule to mediate M3R-dependent EGFR activation using a positive feedback mechanism [10]. Hence, we speculate that increased *Mmp7* expression may be a major contributing factor to bethanechol-stimulated tumorigenesis.

## Conclusions

Collectively, these observations expand our understanding of the molecular mechanisms underlying muscarinic receptor-dependent intestinal tumorigenesis and the roles of cholinergic signaling molecules in mediating this process. We identified key genes that can be targeted simultaneously for maximal anti-tumor effect. This will be the focus of future work.

## Competing interests

The authors have no competing interests to declare.

## Authors’ contributions

ZP carried out the animal studies and QPCR experiments, performed statistical analyses and helped with immunohistochemistry. JH and CD performed histological analyses. JPR participated in tumor counting, measurement and revised the manuscript. GX participated in its design, carried out QPCR experiments, performed statistical analyses and drafted the manuscript. All authors read and approved the final manuscript.

## Pre-publication history

The pre-publication history for this paper can be accessed here:

http://www.biomedcentral.com/1471-2407/13/204/prepub

## Supplementary Material

Additional file 1: Table S1 QPCR Primers. Click here for file

Additional file 2: Figure S1 Animal weight and colon length. A. Body weights of mice fed with either water (control) or water containing bethanechol during the 20-week study. B. Colon length measured at week 20. Results are expressed as mean ± SE from all animals from each group (N = 8 control mice and 7 bethanechol-treated mice). Click here for file
