# Supplementary material for: Cholinergic muscarinic receptor activation augments murine intestinal epithelial cell proliferation and tumorigenesis
Source: BMC Cancer. 2013 Apr 24;13:204. doi: 10.1186/1471-2407-13-204 (PMC3640951; doi:10.1186/1471-2407-13-204)
Supplement: Additional file 1: Table S1 — QPCR Primers. [file 1471-2407-13-204-S1.pdf]

Genes	Forward primer sequence 5' to 3'	Reverse primer sequence 5' to 3'
Mmp1a	CTATGGATCCAGGTTATCCCA	TGTGTTGAACTGATTGGTGAAA
Mmp7	CTGTTCCCGGTACTGTGATG	TCACAGCGTGTTCCTCTTTC
Mmp10	ATGGATAAAGGCTTCCCAAG	TGTGATGATCCTCGGAAGAA
Mmp13	CCGCCTCATAGAAGAGGAAT	TTGTTGGCATGACTCTCACA
Egfr	CGCAAACACAATAAACTGGAA	TCCGAGGAGCATAAAGGATT
Cox2	GCAACAGTAGCATCAAACCG	TTAGTGGAACCATTTCTAGGACAA
Hif1a	TAGCTTCGCAGAATGCTCAG	GAATCCTTTCACTCGTTTCCA
Chrm1	TCTCTGAATGCTGGAAGTAAAGA	GAGACCCTAGATTCAGTCCCA
Chrm2	AAACCGGTCCAACCTGAG	GTCAAGTGGCCAAAGAAACA
Chrm3	AGGGCTGACTACTTAATCTTGGATA	TGCAAGGTCATTGTGACTCTC
Chrm4	CAGCGGAGCAAGACAGAAG	GCACAGACTGATTGGCTGAG
Chrm5	TTAAGCTGCTGCTTCTCTGC	TTTCCAGAGGAGTTGCTAAGG
Myc	CAGCAGCGACTCTGAAGAAG	GACTCCGACCTCTTGGGA
Ccnd1	CAACGCACTTTCTTTCCAGA	GACTCCAGAAGGGCTTCAAT
Gapdh	GGTGAAGGTCGGTGTGAAC	TGATGGCAACAATCTCCACT
