# Supplementary figures and images for: Cholinergic muscarinic receptor activation augments murine intestinal epithelial cell proliferation and tumorigenesis
Source: BMC Cancer. 2013 Apr 24;13:204. doi: 10.1186/1471-2407-13-204 (PMC3640951; doi:10.1186/1471-2407-13-204)

## Slide 1
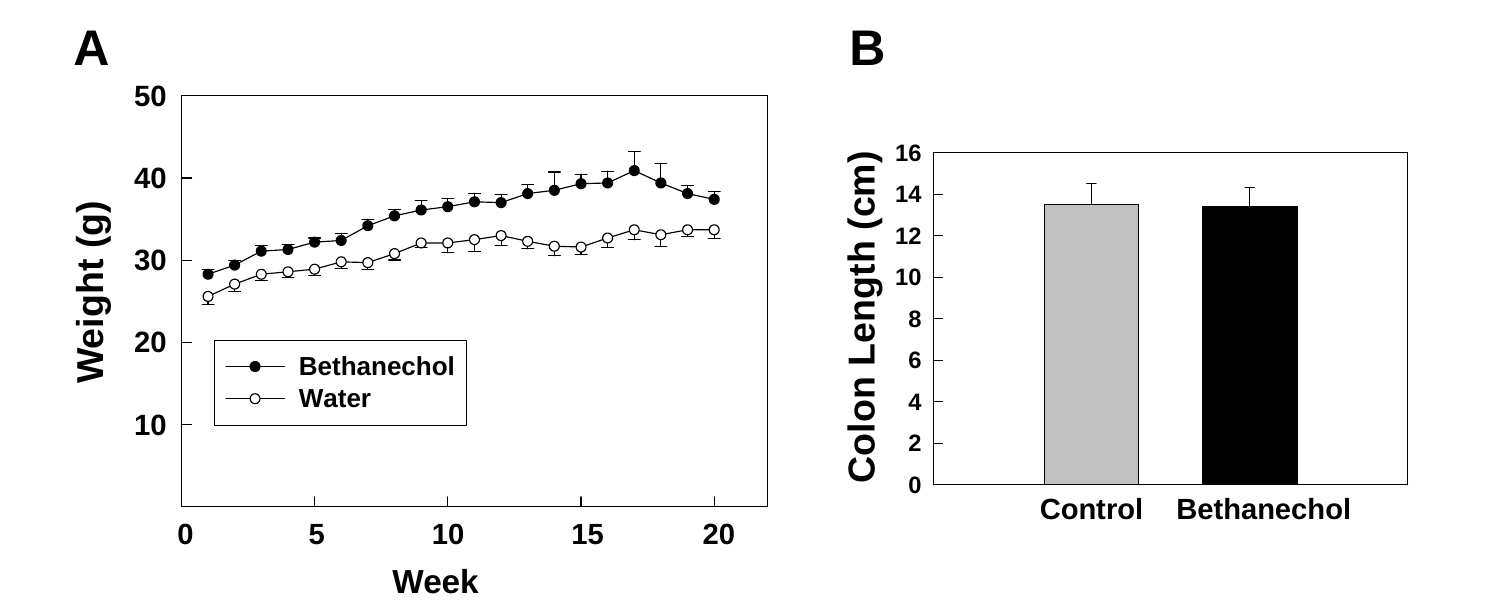

A
B
Weight (g)
Colon Length (cm)
Control Bethanechol
0 5 10 15 20
Week

Supplement: Additional file 2: Figure S1 — Animal weight and colon length. A. Body weights of mice fed with either water (control) or water containing bethanechol during the 20-week study. B. Colon length measured at week 20. Results are expressed as mean ± SE from all animals from each group (N = 8 control mice and 7 bethanechol-treated mice). [file 1471-2407-13-204-S2.ppt]
